# Management of incidentally diagnosed Meckel's diverticulum in pediatric patients: a questionnaire survey and a literature review

**DOI:** 10.3389/fsurg.2026.1719522

**Published:** 2026-05-28

**Authors:** Katarzyna Staniul, Wojciech Biegański, Joanna Tyrchniewicz, Marcin Polok

**Affiliations:** 1 Department of Pediatric Surgery and Urology, University of Zielona Gora, Zielona Góra, Poland; 2 Student Scientific Association of Pediatric Surgery and Urology, Collegium Medicum, University of Zielona Gora, Zielona Góra, Poland

**Keywords:** incidental Meckel's diverticulum, intestinal resection, Meckel diverticulum, Meckel's diveritculum, pediatric surgery, resection

## Abstract

**Introduction:**

The presence of Meckel's diverticulum is an indication for its surgical removal. However, the procedure for treating incidentally found Meckel's diverticulum is controversial. Is its resection tangential to the intestinal wall using, for example, a surgical stapler sufficient? Should wedge/segmental intestinal resection with end-to-end anastomosis be performed? Is primary diverticulectomy always safe, or should the procedure be postponed and classified as an elective?

**Aim:**

The objective of the study was to assess the management of incidentally found Meckel's diverticulum based on a questionnaire survey and a literature review.

**Material and methods:**

A questionnaire survey was sent to pediatric surgeons in Poland. It included 10 questions about steps taken upon the incidental finding of Meckel's diverticulum during appendectomy and the methods of its treatment. The literature review involved searching PubMed. The analysis included 45 articles published in English.

**Results:**

Eighty-six responses were obtained. Sixty-three (61.63%) respondents stated they look for Meckel's diverticulum during each appendectomy. When Meckel's diverticulum is found, 50 (58.14%) respondents excise it during the primary surgery, but in the case of peritonitis symptoms, only 15 (17.44%) respondents choose to remove it. Meckel's diverticulum with a narrow base is excised by wedge or segmental resection by 50 (58.14%) respondents, with a tangential excision with suture closure of the base performed by 35 (40.70%) respondents. According to 76 (88.37%) respondents, Meckel's diverticulum with a wide base is primarily removed by wedge or segmental resection. 21 (24.42%) respondents stated they have heard of complications associated with non-radical Meckel's diverticulum excision and 6 (6.98%) respondents have encountered them in person. Of these 6 cases, 5 involved bleeding from residual ectopic gastric tissue, and 1 case developed symptoms of acute pancreatitis from residual pancreatic tissue.

**Conclusions:**

There is a lack of consensus regarding the management of incidentally diagnosed MD. The decision to proceed with an excision is predicated on the clinical situation. Based on our questionnaire survey, tangential resection is mostly used for long MD with a narrow base. For short MD with a wide base, a safer method seems to be a wedge or rectum section of the intestine. The distinction between the methods results from a high probability of ectopic tissue for MD with a wide base. Postoperative complications occur infrequently, considering all the various operative techniques in use.

## Introduction

Meckel's diverticulum (MD) is a true diverticulum situated on the antimesenteric intestine side about 60 cm from the ileocaecal valve. Its average length is 5 cm (1–3). This congenital defect is caused by an incomplete obliteration of the vitelline duct in the fifth week of embryogenesis. This malformation was first described by Johann Friedrich Meckel in 1598 (1–3). It occurs in 0.3%–2.9% of the population and is most frequently diagnosed intraoperatively during abdominal surgeries for other indications. The presence of MD is an indication for its surgical removal. However, it is controversial which surgical technique—wedge of the adjacent ileum resection, segmental intestinal resection with anastomosis, or tangential excision of the diverticulum—should be selected. Moreover, different surgical methods are used: open, laparoscopic, and hybrid. Furthermore, in some cases, it might be undetermined whether the excision of incidentally discovered MD should be performed as another procedure during one surgery or postponed.

## Objective

The aim of the study was to evaluate ways of managing incidentally found MD in pediatric patients based on a questionnaire survey and a review of the literature.

## Materials and methods

A questionnaire survey with 10 questions on the management of incidentally found MD was sent to pediatric surgeons in Poland. The questions concerned the use of surgical techniques such as intestinal line resection, wedge resection and segmental resection. There were 86 responses received.

For the literature review, PubMed was searched for studies based on the following terms: “Meckel Diverticulum” and “Meckel's Diverticulum.” The analysis included 45 original studies concerning surgical treatment, published in English from 1967 to 2022: prospective studies of children (*n* = 1), prospective studies of adults (*n* = 1), retrospective studies of children (*n* = 14), retrospective studies of both children and adults (*n* = 11), retrospective studies of adults (*n* = 6), and literature reviews (*n* = 12). Of note, no randomized studies were identified. We excluded single case reports and articles without full text.

## Results

Table 1 presents the questions and the respondents’ answers. Sixty-three (61.63%) respondents stated that they look for MD during each appendectomy. When MD is found, 50 (58.14%) respondents excise it during the primary surgery, but with peritonitis symptoms, only 15 (17.44%) respondents decide to remove it. When classifying the removal as deferred, 43 (50%) respondents perform the procedure within 6 months of the primary surgery. MD with a narrow base is removed by wedge or segmental resection by 50 (58.14%) respondents, while 35 (40.70%) respondents perform tangential excision with suture closure of the base (by various techniques). 76 (88.37%) respondents remove a wide-base MD by wedge or segmental resection. Upon finding MD with a demarcated thickening corresponding to ectopic tissue protruding over the intestinal wall, 58 (66.66%) respondents proceed by removing it tangentially. 21 (24.42%) respondents stated that they have heard of complications associated with non-radical excision of MD, including leaving ectopic tissue. Only 6 (6.98%) respondents have encountered it themselves. The most commonly reported complication was bleeding from the ectopic gastric tissue. In a separate case, one patient developed the symptoms of acute pancreatitis after the operation. In a histopathological examination, pancreatic tissue was visualized on the incision line of the diverticulum. The fragment of the intestine was excised during a re-operation. There were no signs of acute pancreatitis after the second procedure.

**Table 1:** Survey results concerning management of incidental Meckel's diverticulum (*N* = 86).

Survey question	Response option	Frequency, % (n)
Do you always look for Meckel's diverticulum during an appendectomy?	Yes	61.63% (53)
	No	26.74% (23)
	It depends	10.46% (9)
When you decide to remove Meckel's diverticulum – do you remove it during primary surgery?	Yes	58.14% (50)
	No	4.65% (4)
	It depends	37.21% (32)
Do you remove Meckel's diverticulum when patient has symptoms of peritonitis?	Yes	17.44% (15)
	No	52.32% (45)
	It depends	30.23% (26)
If you qualify the patient for removal of Meckel's diverticulum at a later date – how long after the first surgery?	Up to 6 months	50.00% (43)
	Over 6 months	17.44% (15)
	Not applicable	32.56% (28)
How do you typically remove a narrow-based Meckel's diverticulum?	In the intestinal line	36.79% (35)
	Wedge or segmental resection	58.14% (50)
	Not applicable	1.16% (1)
How do you typically remove a wide-based Meckel's diverticulum?	In the intestinal line	11.63% (10)
	Wedge or segmental resection	88.37% (76)
	Not applicable	1.16% (1)
If a Meckel's diverticulum is found with clearly palpable ectopic tissue in its bottom, instead of a wedge resection, do you perform a resection in the intestinal line, ensuring macroscopic radicality?	Yes	66.66% (58)
	No	32.56% (28)
Have you experienced or heard about a complication resulting from non-radical removal of Meckel's diverticulum?	No	75.58% (65)
	Yes, heard	16.09% (14)
	Yes, experienced	6.80% (6)
	Yes, read	1.16% (1)
What type of complication occurred?	Bleeding from ectopic gastric tissue	5,81% (5)
	Acute pancreatitis after operation	1,16% (1)

## Discussion

Based on the results of our survey, a majority (61.63%) of pediatric surgeons look for MD during each appendectomy, but its removal depends on the intraoperative signs of peritonitis. Some MD cases qualify for deferred surgery, mostly within 6 months of the primary treatment. In a retrospective analysis, Chen et al. (4) included 53 patients with an incidental finding of MD, of which 2 (3.77%) were cases of postponed resection. Researchers have recommended that for each acute abdominal surgery and appendectomy, surgeons should look for MD and, if found, perform a diverticulectomy (4–6). This type of treatment is recommended based on an enhanced risk of complications associated with symptomatic MD and reinterventions (5–7). Bani-Hani et al. (8) described 4 patients who during previous surgeries had been diagnosed with MD and developed the symptoms associated with it 3–8 years after the primary surgery. On the contrary, Leijonmarck et al. (9) did not describe complications in 26 patients with MD left *in situ* for 7–8 years. When a patient is in a critical medical state and presents symptoms of widespread peritonitis, intestinal obstruction, and/or intestinal perforation not associated with MD, it is recommended to defer the diverticulectomy and to classify it as an elective procedure (5, 6). In these cases, MD should be excised 3–4 months after the primary surgery (5). Ueberrueck et al. (10) recommended that, in the case of gangrene appendicitis, MD should be left *in situ*.

The vast majority of respondents did not have knowledge of and had not encountered complications related to non-radical excision of MD. Indeed, only 6 (6.7%) respondents had experience with such a complication. Of the 6 cases with complications, 5 involved bleeding from residual ectopic tissue of the stomach, and 1 case developed symptoms of acute pancreatitis. The surgical technique used to treat patients with complications was not specified. We did not find cases with similar complications in the literature.

The prevailing complication after the excision of MD is an adhesive intestinal obstruction, which occurs in 5%–10% of adult patients (11). None of the respondents reported similar complications. According to Park et al. (12), none of the complications described after symptomatic or asymptomatic MD resections were associated with diverticulectomy. The risk factors for complications are primarily older age and preoperative diagnosis, for example, obstruction (13–16). In a group of 164 pediatric patients, St-Vil et al. (17) reported 6 cases of postoperative obstruction associated with removal of symptomatic MD and 2 cases of complications for asymptomatic MD (wound infection and intestinal anastomotic leak). Researchers have described cases of adenocarcinoma in asymptomatic MD in adult patients (9, 17–19). The risk associated with carcinogenesis increases with age (16, 20). Among 208 cases of MD in children, Francis et al. (21) described one case of a desmoid tumor. None of the respondents to our questionnaire described a similar case.

Management of asymptomatic, incidentally diagnosed MD is controversial, and there are no clear guidelines. The presence of ectopic tissue in MD is the major determinant of complications (22). Ectopic tissue in MD occurs in 61%–80% of symptomatic cases (2, 12, 23, 24) and only 13%–30% of asymptomatic cases (2, 12, 17). This ectopic tissue is mainly gastric and pancreatic. Ectopic tissue can sometimes be identified on palpation as a thickening, but a high percentage of false-negative results mean that the decision to excise MD should not be based solely on palpation (24–30). Robijn et al. (31) developed a risk assessment scale to assess the likelihood of MD-related complications. It is based on 4 factors: age under 45 years, the male sex, length of > 2 cm, and intestinal adhesions. However, it does not include a palpation assessment of ectopic tissue due to the low specificity and difficulty of assessing it laparoscopically (24, 25, 32). Park et al. (12) observed ectopic tissue upon microscopic examination in 21 out of 100 MD and palpable during resection in 8 of them. In 2 retrospective studies, the authors described the occurrence of palpable ectopic tissue among asymptomatic patients, with histopathological confirmation in 7 out of 25 patients (28%) (17, 33). As a result of the much less frequent prevalence of ectopic tissue in incidentally found MD, many authors recommend a simple diverticulectomy for MD that are short, have narrow base, and lack palpable ectopic tissue (9, 20, 31, 33–35). In our questionnaire survey, 58 (66.67%) respondents indicated that if palpable ectopic tissue is situated only at the MD peak, then they perform tangential excision. Brungardt et al. (15) analyzed 506 adult cases in the American College of Surgeons National Surgical Quality Improvement Program (ACS NSQIP) database and found that diverticulectomy and resection of the small bowel are both appropriate approaches to manage symptomatic MD.

The surgical treatment modality for MD is another issue. As laparoscopy has developed, it is being chosen more frequently. Laparoscopy is limited by an inability to palpate MD for ectopic tissue (22, 24, 25, 31). Redman et al. (35) compared laparoscopy with laparoscopic-assisted resection of MD. They found that laparoscopy is a safe method for managing symptomatic MD, but it entails a 1% risk of leaving residual ectopic tissue. Laparoscopic-assisted diverticulectomy guarantees radical excision of ectopic tissue, reduces costs (it does not utilize a stapler), and lowers the risk of anastomotic leak (4, 13, 35, 36). In a study of a group of 33 children, Shalaby et al. (2) described that short and wide MD with a height-to-diameter (HD) ratio < 1.6 require wedge or segmental resection of the intestine to ensure treatment radicality. In this case, it is best to perform laparotomy or laparoscopic-assisted resection (2, 11, 12, 23, 35). Excision of asymptomatic wide MD is also advised, as there is a higher risk of ectopic tissue and thus a higher risk of complications (23, 24, 35). In a retrospective analysis of 8 patients, Mukai et al. (22) determined the distribution of areas of ectopic tissue in MD based on the HD ratio: They found it only in the distal part of long MD (HD ratio > 1.6), while it occurred in almost all areas of short MD. In a study based on an analysis of the American College of Surgeons National Quality Improvement Project – Pediatric (ACS NSQIP-P) including 681 patients who underwent MD resection, Skertich et al. (37) compared laparoscopic surgery, conversion of laparoscopic to open surgery, and open surgery. This study demonstrated a significantly shorter length of stay in the laparoscopic group (3 vs. 4 days). The laparoscopic approach had similar overall morbidity and operative time as the open approach. The rate of laparoscopic conversion to open surgery was high (31% of laparoscopic cases) and it increased the operative time; however, this conversion did not significantly increase postoperative morbidity (37). Our respondents indicates that they usually remove MD with a narrow base by wedge or segmental resection of the intestine, but some of the respondents ligate MD tangentially using an endoloop, a Roeder loop, a stapler, or even clips. Regarding MD with a wide base, 87% (76) respondents chose wedge or segmental resection of the intestine; the other respondents use a stapler. Of note, there are a lack of published studies with large groups of pediatric patients, most likely because MD are relatively rare in the pediatric population.

## Conclusions

There is a lack of consensus regarding the management of incidentally diagnosed MD. The decision to proceed with an excision is predicated on the clinical situation. Based on our questionnaire survey, tangential resection is mostly used for long MD with a narrow base. For short MD with a wide base, a safer method seems to be a wedge or rectum section of the intestine. The distinction between the methods results from a high probability of ectopic tissue for MD with a wide base. Postoperative complications occur infrequently, considering all the various operative techniques in use.

## References

[1] Tartaglia D Cremonini C Strambi S Ginesini M Biloslavo A Paiano L Incidentally discovered Meckel's diverticulum: should I stay or should I go? ANZ J Surg. (2020) 90(9):1694–9. 10.1111/ans.16189 32783315

[2] Shalaby RY Soliman SM Fawy M Samaha A. Laparoscopic management of Meckel's diverticulum in children. J Pediatr Surg. (2005) 40(3):562–7. 10.1016/j.jpedsurg.2004.11.032 15793736

[3] Palanivelu C Rangarajan M Senthilkumar R Madankumar MV Kavalakat AJ. Laparoscopic management of symptomatic Meckel's diverticula: a simple tangential stapler excision. JSLS. (2008) 12(1):66–70. 18402742 PMC3016022

[4] Chen Q Gao Z Zhang L Zhang Y Pan T Cai D Multifaceted behavior of Meckel's diverticulum in children. J Pediatr Surg. (2018) 53(4):676–81. 10.1016/j.jpedsurg.2017.11.059 29331260

[5] Tauro LF George C Rao BS Martis JJ Menezes LT Shenoy HD. Asymptomatic Meckel's diverticulum in adults: is diverticulectomy indicated? Saudi J Gastroenterol. (2010) 16(3):198–202. 10.4103/1319-3767.65199 20616416 PMC3003224

[6] Matsagas MI Fatouros M Koulouras B Giannoukas AD. Incidence, complications, and management of Meckel's diverticulum. Arch Surg. (1995) 130(2):143–6. 10.1001/archsurg.1995.01430020033003 7848082

[7] Fusco JC Achey MA Upperman JS. Meckel's diverticulum: evaluation and management. Semin Pediatr Surg. (2022) 31(1):151142. 10.1016/j.sempedsurg.2022.151142 35305798

[8] Bani-Hani KE Shatnawi NJ. Meckel's diverticulum: comparison of incidental and symptomatic cases. World J Surg. (2004) 28(9):917–20. 10.1007/s00268-004-7512-3 15593467

[9] Leijonmarck CE Bonman-Sandelin K Frisell J Räf L. Meckel's diverticulum in the adult. Br J Surg. (1986) 73(2):146–9. 10.1002/bjs.1800730225 3484984

[10] Ueberrueck T Meyer L Koch A Hinkel M Kube R Gastinger I. The significance of Meckel's diverticulum in appendicitis–a retrospective analysis of 233 cases. World J Surg. (2005) 29(4):455–8. 10.1007/s00268-004-7615-x 15776296

[11] Onen A Ciğdem MK Oztürk H Otçu S Dokucu AI. When to resect and when not to resect an asymptomatic Meckel's diverticulum: an ongoing challenge. Pediatr Surg Int. (2003) 19(1–2):57–61. 10.1007/s00383-002-0850-z 12721725

[12] Park JJ Wolff BG Tollefson MK Walsh EE Larson DR. Meckel diverticulum: the Mayo Clinic experience with 1476 patients (1950–2002). Ann Surg. (2005) 241(3):529–33. 10.1097/01.sla.0000154270.14308.5f 15729078 PMC1356994

[13] Jung HS Park JH Yoon SN Kang BM Oh BY Kim JW. Clinical outcomes of minimally invasive surgery for Meckel diverticulum: a multicenter study. Ann Surg Treat Res. (2020) 99(4):213–20. 10.4174/astr.2020.99.4.213 33029480 PMC7520228

[14] Zani A Eaton S Rees CM Pierro A. Incidentally detected Meckel diverticulum: to resect or not to resect? Ann Surg. (2008) 247(2):276–81. 10.1097/SLA.0b013e31815aaaf8 18216533

[15] Brungardt JG Cummiskey BR Schropp KP. Meckel's diverticulum: a national surgical quality improvement program survey in adults comparing diverticulectomy and small bowel resection. Am Surg. (2021) 87(6):892–6. 10.1177/0003134820954820 33284028

[16] Lindeman RJ Søreide K. The many faces of Meckel's diverticulum: update on management in incidental and symptomatic patients. Curr Gastroenterol Rep. (2020) 22(1):3. 10.1007/s11894-019-0742-1 31930430

[17] St-Vil D Brandt ML Panic S Bensoussan AL Blanchard H. Meckel's diverticulum in children: a 20-year review. J Pediatr Surg. (1991) 26(11):1289–92. 10.1016/0022-3468(91)90601-O 1812259

[18] Mora-Guzmán I Muñoz de Nova JL Martín-Pérez E. Meckel's diverticulum in the adult: surgical treatment. Acta Chir Belg. (2019) 119(5):277–81. 10.1080/00015458.2018.1503391 30259784

[19] DiGiacomo JC Cottone FJ. Surgical treatment of Meckel's diverticulum. South Med J. (1993) 86(6):671–5. 10.1097/00007611-199306000-00017 8506491

[20] Root GT Baker CP. Complications associated with Meckel's diverticulum. Am J Surg. (1967) 114(2):285–8. 10.1016/0002-9610(67)90385-6 6028991

[21] Francis A Kantarovich D Khoshnam N Alazraki AL Patel B Shehata BM. Pediatric Meckel's diverticulum: report of 208 cases and review of the literature. Fetal Pediatr Pathol. (2016) 35(3):199–206. 10.3109/15513815.2016.1161684 27064958

[22] Mukai M Takamatsu H Noguchi H Fukushige T Tahara H Kaji T. Does the external appearance of a Meckel's diverticulum assist in choice of the laparoscopic procedure? Pediatr Surg Int. (2002) 18(4):231–3. 10.1007/s003830100663 12021967

[23] Hansen CC Søreide K. Systematic review of epidemiology, presentation, and management of Meckel's diverticulum in the 21st century. Medicine (Baltimore). (2018) 97(35):e12154. 10.1097/MD.0000000000012154 30170459 PMC6392637

[24] Varcoe RL Wong SW Taylor CF Newstead GL. Diverticulectomy is inadequate treatment for short Meckel's diverticulum with heterotopic mucosa. ANZ J Surg. (2004) 74(10):869–72. 10.1111/j.1445-1433.2004.03191.x 15456435

[25] Chan KW Lee KH Mou JW Cheung ST Tam YH. Laparoscopic management of complicated Meckel's diverticulum in children: a 10-year review. Surg Endosc. (2008) 22(6):1509–12. 10.1007/s00464-008-9832-0 18322735

[26] Schier F Hoffmann K Waldschmidt J. Laparoscopic removal of Meckel's diverticula in children. Eur J Pediatr Surg. (1996) 6(1):38–9. 10.1055/s-2008-1066466 8721178

[27] Cullen JJ Kelly KA Moir CR Hodge DO Zinsmeister AR Melton LJ 3rd. Surgical management of Meckel's diverticulum. An epidemiologic, population-based study. Ann Surg. (1994) 220(4):564–9. 10.1097/00000658-199410000-00014 7944666 PMC1234434

[28] Michas CA Cohen SE Wolfman EF Jr. Meckel's diverticulum: should it be excised incidentally at operation? Am J Surg. (1975) 129(6):682–5. 10.1016/0002-9610(75)90345-1 1079410

[29] Diamond T Russell CF. Meckel's diverticulum in the adult. Br J Surg. (1985) 72(6):480–2. 10.1002/bjs.1800720625 3874676

[30] Blouhos K Boulas KA Tsalis K Barettas N Paraskeva A Kariotis I Meckel's diverticulum in adults: surgical concerns. Front Surg. (2018) 5:55. 10.3389/fsurg.2018.00055 30234126 PMC6129587

[31] Robijn J Sebrechts E Miserez M. Management of incidentally found Meckel's diverticulum a new approach: resection based on a risk score. Acta Chir Belg. (2006) 106(4):467–70. 10.1080/00015458.2006.11679933 17017710

[32] Ezekian B Leraas HJ Englum BR Gilmore BF Reed C Fitzgerald TN Outcomes of laparoscopic resection of Meckel's diverticulum are equivalent to open laparotomy. J Pediatr Surg. (2019) 54(3):507–10. 10.1016/j.jpedsurg.2018.03.010 29661575

[33] Karaman A Karaman İ Çavuşoağlu YH Erdoağan D Aslan MK. Management of asymptomatic or incidental Meckels diverticulum. Indian Pediatr. (2010) 47(12):1055–7. 10.1007/s13312-010-0176-1 19671945

[34] Fa-Si-Oen PR Roumen RM van Uchelen C A F. Complications and management of Meckel's Diverticulum–a review. Eur J Surg. (1999) 165(7):674–8. 10.1080/11024159950189735 10452262

[35] Redman EP Mishra PR Stringer MD. Laparoscopic diverticulectomy or laparoscopic-assisted resection of symptomatic meckel diverticulum in children? A systematic review. Pediatr Surg Int. (2020) 36(8):869–74. 10.1007/s00383-020-04673-5 32436063

[36] Lee KH Yeung CK Tam YH Ng WT Yip KF. Laparascopy for definitive diagnosis and treatment of gastrointestinal bleeding of obscure origin in children. J Pediatr Surg. (2000) 35(9):1291–3. 10.1053/jpsu.2000.9299 10999681

[37] Skertich NJ Ingram MC Grunvald MW Williams MD Ritz E Shah AN Outcomes of laparoscopic versus open resection of Meckel's diverticulum. J Surg Res. (2021) 264:362–7. 10.1016/j.jss.2021.02.028 33848834

